# Age-Related Alterations of Cerebral Autoregulation

**DOI:** 10.3390/life15111669

**Published:** 2025-10-27

**Authors:** Anna Ungvari, Attila Kállai, Levente Stankovics, Dominika Lendvai-Emmert, Rafal Gulej, Eva Pal, Roland Patai, Boglarka Csik, Mónika Fekete, Ágnes Lipecz, Tamás Csípő, Zoltán Benyó, Anna Csiszar, Peter Toth

**Affiliations:** 1 Institute of Preventive Medicine and Public Health, Semmelweis University, 1089 Budapest, Hungary; ungann2004@gmail.com (A.U.);; 2 Fodor Center for Prevention and Healthy Aging, Semmelweis University, 1089 Budapest, Hungary; 3 Health Sciences Division, Doctoral College, Semmelweis University, 1089 Budapest, Hungary; 4 Department of Neurosurgery, Medical School, University of Pecs, 7624 Pecs, Hungary; 5 Vascular Cognitive Impairment, Neurodegeneration and Healthy Brain Aging Program, Department of Neurosurgery, University of Oklahoma Health Sciences Center, Oklahoma City, OK 73104-3609, USA; 6 International Training Program in Geroscience, Doctoral College-Health Sciences Division/Institute of Preventive Medicine and Public Health, Semmelweis University, 1089 Budapest, Hungary; 7 Institute of Translational Medicine, Semmelweis University, 1094 Budapest, Hungary; 8 HUN-REN-SU Cerebrovascular and Neurocognitive Diseases Research Group, 1094 Budapest, Hungary

**Keywords:** cerebral blood flow, ageing, hypertension, cerebrovascular disease

## Abstract

Intact regulation of cerebral blood flow (CBF) is essential for preserving cognitive function and reducing the risk of cerebrovascular events, particularly in the aging population. Autoregulation of CBF is one of the fundamental mechanisms that ensure constant supply for brain tissue by maintaining relatively stable perfusion despite fluctuations in systemic blood pressure. It also acts as a critical protective mechanism, shielding the fragile cerebral microcirculation from potentially harmful pressure fluctuations and hence excessive pulsatility. The loss or attenuation of this protective mechanism with aging or disease increases the vulnerability of the microvasculature to structural damage, blood–brain barrier (BBB) disruption, and the development of cerebral small vessel disease. This mini-review summarizes current understanding of how aging affects cerebral autoregulation, highlighting underlying mechanisms, clinical consequences, and potential strategies to preserve cerebrovascular health in older adults.

## 1. Introduction

The human brain is critically dependent on a constant supply of oxygen and nutrients, which is ensured by precise regulation of cerebral blood flow (CBF). Cerebral autoregulation refers to the intrinsic ability of regulation of CBF to maintain relatively stable perfusion of cerebral tissue despite fluctuations in systemic blood pressure [1]. Importantly, cerebral autoregulation also serves as a critical protective mechanism, shielding the fragile cerebral microcirculation from potentially harmful pressure fluctuations and hence excessive pulsatility [2]. By preventing the direct transmission of pulsatile or excessive pressure waves into the microvascular bed, autoregulation protects capillaries, arterioles, and the blood–brain barrier (BBB) from mechanical stress and injury [3]. The loss or attenuation of this protective mechanism with aging or disease increases the vulnerability of the microvasculature to structural damage, BBB disruption, and the development of cerebral small vessel disease [4]. Thus, intact autoregulation is fundamental not only for maintaining adequate perfusion but also for preserving the structural and functional integrity of the cerebral microcirculation [2].

Intact regulation of CBF is essential for preserving cognitive function and reducing the risk of cerebrovascular events, particularly in the aging population, where both vascular and neural systems undergo profound structural and functional changes [5]. The process of aging is associated with increased vulnerability to cerebrovascular insults, yet the role of impaired cerebral autoregulation in this context is often underrecognized [6,7].

This mini-review summarizes current understanding of how aging affects cerebral autoregulation, highlighting underlying mechanisms, clinical consequences, and potential strategies to preserve cerebrovascular health in older adults.

## 2. Mechanisms of Cerebral Autoregulation

Under physiological conditions, autoregulation operates effectively within a mean arterial pressure (MAP) range of approximately 50 to 150 mmHg [3]. Within these limits, the cerebrovascular system dynamically adjusts vascular resistance to counterbalance changes in perfusion pressure, ensuring stable delivery of oxygen and nutrients to the brain and protecting the fragile microcirculation from pressure-induced damage [4].

Importantly, a distinction is made between static autoregulation [8], which reflects the steady-state relationship between MAP and CBF, and dynamic autoregulation [9,10,11], which describes the rapid adjustments of cerebrovascular resistance to transient blood pressure fluctuations. Dynamic autoregulation is often more sensitive to early vascular dysfunction and is therefore particularly relevant when considering age-related impairments [12].

This complex regulatory process is governed by several interacting mechanisms. One of the most immediate contributors is the myogenic response, whereby vascular smooth muscle cells contract or relax in direct response to changes in transmural pressure [13]. This rapid adjustment stabilizes blood flow on a beat-to-beat basis and protects the delicate cerebral microvasculature from excessive pressure fluctuations [14]. The rapid myogenic adjustment of vascular tone is mainly controlled by cellular mechanisms in vascular smooth muscle cells, which convert mechanical stimuli into contractile responses. A key aspect of this process is the activation of mechanosensitive ion channels, especially voltage-gated calcium channels (VGCCs), which are essential for triggering vasoconstriction [15].

When transmural pressure increases, the resulting stretch of the vascular wall activates mechanosensors embedded in the smooth muscle cell membrane. This mechanical stimulus triggers the opening of L-type voltage-gated calcium channels (Cav1.2) [16], allowing extracellular calcium to enter the cytoplasm. An increase in intracellular calcium concentration acts as the key signal to start smooth muscle contraction. It activates the calcium–calmodulin complex and stimulates myosin light chain kinase (MLCK), which phosphorylates myosin light chains and enables actin–myosin cross-bridge cycling [17].

In addition to VGCCs, transient receptor potential (TRP) channels, particularly those of the TRPC and TRPV subfamilies, contribute to myogenic constriction. These non-selective cation channels are sensitive to mechanical stretch and facilitate calcium influx or membrane depolarization, which in turn enhances the activation of VGCCs and amplifies the contractile response [18].

The myogenic response is further modulated by a network of intracellular signaling pathways, including the activation of Rho-associated kinase (ROCK) and protein kinase C (PKC) [19]. These kinases promote calcium sensitization, a process whereby vascular smooth muscle contraction is maintained or enhanced independently of further increases in intracellular calcium levels. Specifically, ROCK inhibits myosin light chain phosphatase (MLCP), thereby sustaining myosin light chain phosphorylation and augmenting vasoconstriction [16].

Together, these coordinated molecular events—activation of mechanosensitive ion channels, calcium influx through VGCCs and TRP channels, and kinase-mediated calcium sensitization—allow vascular smooth muscle cells to adjust vascular tone rapidly and effectively. This adjustment of tone changes vascular resistance in response to pressure fluctuations, helping to maintain stable cerebral perfusion [20].

In addition to intrinsic vascular responses, neurogenic control also plays a role, particularly in larger cerebral vessels. Sympathetic and parasympathetic innervation modulates vascular tone, providing an additional layer of regulation that complements local mechanisms [21].

The brain’s ability to match blood flow to metabolic demands is further supported by metabolic regulation. Changes in local concentrations of carbon dioxide, pH, and metabolites such as adenosine lead to adjustments in arteriolar diameter, ensuring that neuronal activity is matched by an adequate supply of oxygen and nutrients in a negative feedback manner [22,23].

Endothelial function represents another crucial component of cerebral autoregulation. The vascular endothelium fine-tunes vascular tone by releasing vasoactive substances, including nitric oxide (NO), prostanoids, and endothelin-1 [24,25,26]. These mediators orchestrate the delicate balance between vasodilation and vasoconstriction, contributing to the dynamic stability of CBF.

In the setting of chronic hypertension, the cerebrovascular system undergoes structural and functional adaptations designed to preserve autoregulatory capacity [27]. These include remodeling of resistance vessels, alterations in prostanoid signaling pathways, and the involvement of TRP channels and calcium-dependent signaling cascades [26,28,29]. While such adaptations may initially help maintain CBF stability, they can also render the system more susceptible to dysregulation, a vulnerability that becomes particularly pronounced with advancing age [29].

## 3. Age-Related Changes in Cerebral Autoregulation

### 3.1. Structural Changes Contributing to Impaired Autoregulation

Aging is associated with progressive stiffening of large arteries [30,31,32], degradation of elastin fibers, and increased transmission of pulsatile pressure to the cerebral microcirculation. These changes diminish vascular compliance, reducing the ability of vessels to buffer pressure fluctuations and compromising the autoregulatory reserve [33].

In the context of hypertension, aging alters the normal adaptive remodeling of cerebral vessels. Studies suggest that the ability to remodel appropriately is diminished in older mammals, with dysregulated prostanoid signaling, TRP channel function, and impaired calcium signaling contributing to reduced vascular adaptability [29].

### 3.2. Functional Impairments

Functional assessments of cerebral autoregulation consistently demonstrate age-related attenuation, particularly in dynamic autoregulatory responses, which are essential for stabilizing CBF during rapid or transient blood pressure changes [34,35]. Both older individuals and aged laboratory animals exhibit weakened myogenic responses of cerebral resistance vessels, limiting the vasculature’s ability to constrict or dilate in response to perfusion pressure fluctuations. This impairment in cerebrovascular autoregulation contributes to diminished cerebral blood flow regulation with aging, increasing vulnerability to cerebral hypoperfusion during hemodynamic stress. While detailed quantitative measures such as transfer-function gain, phase, and autoregulatory index values vary across studies and methodologies, this decline in myogenic responsiveness is a consistent feature of cerebral vascular aging [36]. This diminished responsiveness compromises the brain’s capacity to buffer hemodynamic stress, increasing vulnerability to both hypoperfusion and hyperperfusion [2].

Moreover, age-related endothelial dysfunction, characterized by reduced NO bioavailability, increased endothelin-1 production, and heightened oxidative stress, further impairs the fine-tuning of cerebrovascular tone [37]. The endothelium’s ability to dynamically modulate vasodilation and vasoconstriction is essential for effective autoregulation, and its deterioration with aging undermines this regulatory capacity.

In addition to these baseline functional impairments, the adaptive response of the cerebrovasculature to chronic hypertension is also compromised in aging [29]. Under normal conditions, cerebral vessels undergo structural and functional remodeling in response to sustained hypertension, shifting the autoregulatory curve to maintain stable CBF at higher perfusion pressures [38]. However, aging impairs this protective adaptation. Older individuals show inadequate vascular remodeling and altered signaling pathways—including dysregulated prostanoid production, impaired TRP channel function, and disrupted calcium handling—that limit the ability of the cerebral vasculature to effectively compensate for chronic hypertension [29]. As a result, hypertensive older adults are at increased risk for cerebrovascular injury [32,39], as the protective autoregulatory range narrows and the brain becomes more susceptible to both hypo- and hyperperfusion-related damage.

Additionally, neurovascular coupling [40,41], the tightly regulated increase in CBF in response to neuronal activity, is often disrupted with aging. Impaired signaling between neurons, astrocytes, and endothelial cells diminishes the ability of the cerebrovasculature to meet local metabolic demands, further contributing to functional cerebrovascular insufficiency and cognitive decline [42].

Collectively, these functional impairments—including diminished dynamic autoregulation, endothelial dysfunction, inadequate adaptation to hypertension, and disrupted neurovascular coupling—converge to compromise cerebrovascular resilience in older adults, increasing the risk of cognitive impairment and cerebrovascular disease [43].

### 3.3. Emerging Role of Cellular and Molecular Aging Mechanisms

By definition, age-related functional decline must involve the fundamental biological mechanisms of aging. The conceptual framework provided by the hallmarks of aging offers a unifying perspective to understand the molecular drivers of age-associated cerebrovascular dysfunction [44,45,46,47]. These hallmarks include genomic instability, telomere attrition, epigenetic alterations [48], loss of proteostasis, deregulated nutrient sensing, mitochondrial dysfunction [49,50], cellular senescence [51], stem cell exhaustion, altered intercellular communication, heightened state of inflammation [50] and increased oxidative stress [52,53].

In the context of cerebral autoregulation and dysregulation of CBF, cellular senescence plays a central role, particularly within the cerebrovascular endothelium. Senescent endothelial cells lose their proliferative capacity and exhibit profound alterations in function, including increased oxidative stress, diminished NO bioavailability, and the secretion of pro-inflammatory and matrix-degrading factors—a profile collectively referred to as the senescence-associated secretory phenotype (SASP) [54,55]. These changes disrupt vascular homeostasis and blunt the dynamic regulation of vascular tone, undermining the brain’s ability to maintain stable perfusion. Moreover, accumulating evidence indicates that increased vascular senescence contributes to several pathological features associated with cerebrovascular aging. Specifically, endothelial senescence has been linked to the development of CMHs, subtle yet clinically significant markers of microvascular fragility and impaired hemodynamic control [56]. Senescence is also implicated in BBB disruption [57], a hallmark of cerebrovascular dysfunction that further compromises brain homeostasis and increases susceptibility to neurodegeneration. In addition, neurovascular coupling (NVC) impairment [58], which reflects the inability of cerebral blood vessels to adjust perfusion in response to neuronal activity, has been associated with vascular aging and senescence-driven endothelial dysfunction [57]. These interconnected processes highlight the vulnerability of the aging cerebrovascular system and underscore the importance of studying cerebral autoregulation and vascular smooth muscle cell (VSMC) function as critical determinants of brain health. Understanding how senescence disrupts both endothelial and smooth muscle components of the vascular wall is essential for identifying therapeutic strategies aimed at preserving autoregulatory capacity and reducing the risk of cerebrovascular pathology in aging.

Another key hallmark implicated in age-related cerebrovascular impairment is mitochondrial dysfunction [59]. Age-associated decline in mitochondrial function leads to excessive production of reactive oxygen species (ROS), which exacerbates oxidative stress, impairs endothelial signaling, and promotes vasoconstriction [60]. This oxidative environment compromises NO signaling, a critical determinant of vascular tone regulation, and amplifies vascular inflammation, thereby destabilizing cerebrovascular homeostasis [61]. Emerging evidence further links mitochondrial dysfunction to endothelial dysfunction, which represents an early and central feature of cerebrovascular aging. Mitochondrial impairment within endothelial cells disrupts energy production, increases oxidative burden, and compromises endothelial barrier integrity [62]. Consequently, mitochondrial dysfunction has been implicated in BBB disruption, a key pathological feature contributing to increased vascular permeability and susceptibility to neuroinflammation in the aging brain [63]. In addition, mitochondrial dysfunction is increasingly recognized as a contributor to NVC impairment [62,63]. This deficit impairs the brain’s ability to match metabolic demand with adequate perfusion, contributing to cognitive decline and functional brain deficits in older adults [64]. While mitochondrial dysfunction has been strongly linked to endothelial dysfunction, BBB breakdown, and impaired NVC, its direct role in the pathogenesis of CMHs remains less clearly defined [65]. Further research is warranted to elucidate whether mitochondrial decline contributes to autoregulatory dysfunction. For example, traumatic brain injury-induced excessive production of mitochondria derived H2O2 was shown to impair myogenic constriction of cerebral vessels through TRPV4-dependent activation of BK Ca channels [66].

Chronic low-grade inflammation, or “inflammaging”, represents a systemic manifestation of altered intercellular communication, another hallmark of aging. In the cerebral vasculature, this persistent inflammatory state further destabilizes autoregulatory mechanisms by promoting endothelial activation, vascular stiffness, and maladaptive remodeling [67,68]. In addition to the cell-intrinsic mechanisms outlined above, cell non-autonomous processes play a fundamental role in the age-related decline of cerebrovascular function. One of the most extensively studied systemic factors contributing to vascular aging is the progressive decline in circulating levels of insulin-like growth factor-1 (IGF-1) [69]. IGF-1 is a key mediator of vascular maintenance and repair, exerting pleiotropic protective effects on endothelial and smooth muscle cells [69]. The age-associated reduction in IGF-1 production, primarily due to diminished growth hormone (GH) signaling and hepatic output, represents a hallmark of disrupted intercellular communication—a core pillar of the aging process. Experimental studies in mouse models have provided compelling evidence for the critical role of IGF-1 in maintaining cerebrovascular health [69,70,71]. Mice with reduced circulating IGF-1 levels exhibit impaired cerebral autoregulation, characterized by diminished myogenic responses and blunted vascular reactivity to pressure fluctuations. Specifically, the autoregulatory range in IGF-1 deficient mice was narrower (80–140 mmHg) compared to controls (80–170 mmHg), indicating reduced capacity to maintain stable cerebral blood flow across a range of pressures. Although transfer-function gain and phase values were not directly reported, pressure-diameter relationships showed significantly impaired myogenic tone development in IGF-1 deficient hypertensive mice, reflecting marked autoregulatory dysfunction [71]. This impairment compromises the brain’s ability to buffer systemic blood pressure changes, increasing susceptibility to hypoperfusion and microvascular damage. Importantly, the adaptive response to chronic hypertension is also disrupted in the setting of IGF-1 deficiency, as vascular remodeling and functional adjustments required to maintain CBF at elevated perfusion pressures are impaired [71]. The inability to mount an effective hypertensive adaptation exacerbates cerebrovascular vulnerability in aged individuals with comorbid hypertension, a common clinical scenario. IGF-1 deficiency also contributes to endothelial dysfunction, heightened oxidative stress, and increased vascular inflammation [72]. These alterations mirror the endothelial changes observed in aged individuals and synergize with other hallmarks of aging, such as mitochondrial dysfunction and cellular senescence [56,65], to destabilize cerebrovascular homeostasis. In line with these mechanisms, animal studies have shown that IGF-1 deficiency exacerbates BBB disruption [73], a key pathological feature contributing to increased vascular permeability, neuroinflammation, and neuronal injury. Moreover, reduced IGF-1 signaling has been linked to a higher burden of CMHs, further highlighting the role of systemic growth factor deficiency in promoting microvascular fragility and structural damage in aging [74,75]. Findings obtained in heterochronic parabiosis models further emphasize that age-related cerebrovascular dysregulation cannot be fully understood without considering the contribution of cell non-autonomous mechanisms [76], particularly the decline in circulating IGF-1 [77]. Restoring systemic trophic support represents a promising avenue for preserving CBF regulation, protecting microvascular integrity, and mitigating the progression of cerebrovascular pathology in the aging brain.

Together, these interconnected aging hallmarks provide a mechanistic basis for the decline in cerebral autoregulation with advancing age. Importantly, they also identify potential targets for therapeutic intervention aimed at vascular rejuvenation. Strategies that reverse endothelial senescence [56], mitigate mitochondrial dysfunction, or suppress chronic inflammation may hold promise for restoring autoregulatory capacity and protecting cerebrovascular health in older adults. Understanding these processes through the lens of fundamental aging biology is essential for the development of effective interventions to preserve brain function across the lifespan.

## 4. Consequences of Impaired Autoregulation in Aging

Impaired cerebral autoregulation increases the brain’s vulnerability to both hypoperfusion and hyperperfusion, creating conditions that promote microvascular damage. In older adults with compromised autoregulatory capacity, even modest fluctuations in systemic blood pressure can translate into significant alterations in CBF, exceeding the adaptive capacity of the microcirculation [2,3,4]. During hypotensive episodes, this vulnerability manifests as cerebral hypoperfusion, which reduces oxygen and nutrient delivery to the brain and increases the risk of ischemic injury, particularly in wat00ershed regions [2].

Conversely, loss of effective autoregulatory buffering exposes the fragile cerebral microvasculature to damaging surges in blood pressure during hypertensive events [2]. These exaggerated CBF fluctuations impose significant mechanical stress on the capillary and arteriolar networks, which can disrupt the BBB and increase vascular permeability [78]. Over time, this cumulative microvascular injury contributes to the development of CMHs—small, often asymptomatic foci of hemosiderin deposition detectable by susceptibility-weighted imaging (SWI) on MRI [79].

CMHs are increasingly recognized as radiological markers of microvascular fragility and have been strongly linked to aging, hypertension, and cerebral small vessel disease [80]. They predominantly localize to deep brain regions, cortical-subcortical junctions, and lobar territories, depending on the underlying vascular pathology [79,81]. Importantly, CMHs are not merely incidental findings but are associated with cognitive impairment, gait disturbances, and an elevated risk of intracerebral hemorrhage, particularly in patients with mixed cerebrovascular pathology [82,83,84]. Beyond their role as imaging biomarkers, CMHs represent focal sites of brain tissue damage, where the leakage of blood products into the surrounding parenchyma triggers neuroinflammation, oxidative stress, and neuronal injury. Repeated or cumulative CMHs can disrupt neural networks, impair brain connectivity, and contribute to progressive functional decline, reinforcing their significance as both markers and mediators of brain damage in aging and cerebrovascular disease [82,83].

The pathogenesis of CMHs in the context of impaired autoregulation is thought to involve several converging mechanisms [85]. Chronic exposure to hemodynamic stress weakens the structural integrity of small vessels, promoting microaneurysm formation, perivascular inflammation, and degeneration of vascular smooth muscle cells [86]. Age-related endothelial dysfunction and increased oxidative stress further exacerbate vessel wall fragility, impair repair mechanisms, and compromise BBB integrity [53]. In this vulnerable state, transient hypertensive spikes or orthostatic blood pressure variability—common in older adults—can precipitate microvascular rupture, resulting in CMH formation [87]. Given the strong association between CMHs and adverse neurological outcomes [88,89], maintaining effective cerebral autoregulation is critical for protecting microvascular integrity in the aging brain. Understanding how age-related decline in autoregulatory function contributes to CMH development not only provides insight into the mechanisms of vascular cognitive impairment but also highlights potential therapeutic targets aimed at preserving cerebrovascular resilience and reducing the burden of microvascular injury [90].

In addition to these well-established consequences, orthostatic hypotension (OH) represents a clinically relevant manifestation of impaired cerebral autoregulatory capacity in older adults [91]. OH, defined as a significant drop in blood pressure upon standing, is prevalent in aging populations and is often exacerbated by coexisting hypertension, autonomic dysfunction, or polypharmacy [92]. In healthy individuals, intact cerebral autoregulation rapidly compensates for transient reductions in perfusion pressure, maintaining stable CBF during postural changes [2]. However, with advancing age and autoregulatory decline, this compensatory response is reduced, increasing the likelihood of cerebral hypoperfusion during episodes of OH [2,3]. Importantly, recurrent or prolonged cerebral hypoperfusion associated with OH has been linked to cognitive impairment, falls [93], and an elevated risk of ischemic events, particularly in individuals with existing cerebrovascular disease or compromised microvascular integrity. In the context of aging and small vessel disease, the brain’s diminished ability to buffer blood pressure fluctuations may amplify the adverse effects of OH, contributing to progressive white matter damage, microinfarcts, and functional decline [94]. Given the high prevalence of OH in older adults and its potential to exacerbate cerebrovascular injury [92], assessment and management of OH should be considered an integral component of cerebrovascular risk reduction strategies, particularly in individuals with known impaired autoregulation or evidence of microvascular disease.

The deterioration of cerebral autoregulation has been implicated in the pathogenesis of vascular cognitive impairment and dementia (VCID), where chronic hypoperfusion and microvascular dysfunction play central roles [95,96]. Moreover, impaired autoregulation may interact with neurodegenerative processes in mixed dementia, where both VCID and Alzheimer’s disease (AD) pathology coexist [97]. In the development of VCID white matter lesions (WMLs) and lacunar infarcts most likely play critical role. Probably not directly related to autoregulatory dysfunction, white matter hyperintensities, the most common neuroimaging manifestation of cerebral small vessel disease, consistently predict cognitive decline and dementia across diverse populations [98]. These lesions particularly impair executive function and processing speed, reflecting disruption of frontal-subcortical circuits that are essential for complex cognitive tasks. Lacunar infarcts, small subcortical strokes affecting deep brain structures, contribute to cognitive decline despite their modest size, with studies showing that approximately 37% of patients develop mild cognitive impairment or dementia following lacunar stroke [99]. The cognitive impact appears disproportionate to lesion size because lacunar infarcts often occur in the context of widespread cerebral small vessel disease, creating a cumulative burden of microvascular damage. Both WMLs and lacunar infarcts disrupt white matter microstructural integrity, as demonstrated by diffusion tensor imaging studies showing that the count of lacunar infarcts and diffusivity changes in normal-appearing white matter are independent predictors of executive dysfunction [100]. The underlying pathophysiology involves chronic hypoperfusion, blood–brain barrier disruption, and inflammatory responses that lead to demyelination and axonal damage in vulnerable deep white matter regions, ultimately compromising the neural networks essential for cognitive function. As mentioned above, the bidirectional interaction between vascular factors and AD represents a complex, synergistic relationship where cerebrovascular dysfunction both contributes to and results from AD pathology [101]. Vascular dysfunction appears early in AD pathogenesis, often preceding cognitive symptoms and traditional biomarkers, with chronic cerebral hypoperfusion and blood–brain barrier disruption creating conditions that promote amyloid-β (Aβ) accumulation in brain tissue [102]. Conversely, Aβ deposits exert direct neurotoxic effects on cerebral blood vessels, particularly through cerebral amyloid angiopathy (CAA), where Aβ40 and Aβ42 accumulate in vessel walls, causing smooth muscle cell degeneration, vessel wall weakening, and impaired cerebrovascular autoregulation [103,104]. This creates a “two-hit” vascular hypothesis, where initial microvascular insults (hit 1) lead to Aβ-independent neuronal dysfunction and BBB disruption, followed by Aβ-dependent vascular toxicity (hit 2) that creates a positive feedback loop of progressive cerebrovascular damage. Recent evidence demonstrates that vascular risk factors and cerebral small vessel disease severity are directly linked to amyloid deposition and downstream tau pathology, suggesting that cardiovascular health interventions may represent important targets for AD prevention. The clinical significance is substantial, as studies show that up to 50% of dementia cases, including AD, may have a vascular component, emphasizing that effective AD treatment strategies must address both neurodegenerative and cerebrovascular pathways simultaneously.

Interestingly, autoregulatory dysfunction may contribute to the development of age-related disorders of cerebrospinal fluid circulation. Accordingly, normal pressure hydrocephalus (NPH) involves complex vascular mechanisms that extend far beyond simple mechanical compression of brain tissue. The pathophysiology centers on impaired cerebrovascular autoregulation, where studies consistently demonstrate reduced cerebral blood flow (approximately 20–28% below normal) coupled with preserved but unused autoregulatory reserve, creating an apparent paradox where the ischemic brain fails to utilize available compensatory mechanisms [105]. Vascular compliance abnormalities play a central role, with NPH patients showing significantly reduced compliance in the superior sagittal sinus territory compared to healthy controls, leading to altered arteriovenous delay patterns and restricted cerebrospinal fluid outflow [106]. The “two-hit” vascular hypothesis suggests that vascular risk factors (hypertension, diabetes, white matter lesions) predispose to NPH development, with population-based studies demonstrating strong associations between these cardiovascular comorbidities and both clinical and imaging features of NPH [107,108]. Furthermore, recent modeling studies indicate that NPH involves balanced increases in both arterial and venous resistance, with the cerebral blood flow being actively limited by arteriolar constriction rather than passive compression, and this resistance pattern can be partially reversed following shunt surgery [109]. The vascular dysfunction creates a periventricular watershed region with maximal blood flow reduction occurring approximately 9 mm from the ventricular wall, explaining the characteristic pattern of white matter damage and the preferential vulnerability of deep brain structures in NPH [110].

Autoregulatory dysfunction and enlarged perivascular spaces (EPVS) are also interrelated phenomena. Impaired autoregulation can increase the transmission of blood pressure fluctuations to small vessels, promoting endothelial dysfunction and diminished perivascular clearance, factors that further contribute to EPVS formation [111]. Studies have demonstrated that patients with severe basal ganglia EPVS have higher critical closing pressure—a dynamic parameter that also reflects autoregulatory reserve—compared to those with fewer EPVS [112].

Finally, in the context of acute cerebrovascular events such as stroke, impaired autoregulation increases susceptibility to poor outcomes, including larger infarct size and higher risk of hemorrhagic transformation following reperfusion [113,114,115].

An often-overlooked consequence of impaired cerebral autoregulation is its detrimental impact on interhemispheric blood flow compensation following unilateral carotid artery occlusion. Under physiological conditions, intact autoregulatory mechanisms and a functional circle of Willis enable redistribution of blood flow from the contralateral hemisphere to maintain perfusion in territories affected by carotid stenosis or occlusion. However, with advancing age and associated vascular dysfunction, these compensatory pathways become increasingly compromised [33]. Impaired autoregulation blunts the ability of cerebral vessels in the contralateral hemisphere to appropriately dilate in response to increased demand, limiting effective collateral recruitment [116].

This dysfunctional compensatory response is of particular relevance during vascular interventions such as carotid artery stenting (CAS) or carotid endarterectomy (CEA), where temporary or prolonged alterations in perfusion dynamics can occur [117]. In older adults with pre-existing cerebrovascular aging and impaired autoregulation, these procedures carry a higher risk of inadequate interhemispheric flow redistribution, leaving vulnerable brain regions susceptible to hypoperfusion, ischemia, and subsequent neurological complications [118]. Moreover, in the setting of incomplete or hypoplastic components of the circle of Willis—a condition prevalent in a substantial portion of the population [119]—the reliance on intact autoregulatory capacity for adequate collateral flow becomes even more critical. Therefore, understanding the interplay between aging, autoregulatory decline, and interhemispheric perfusion is essential for optimizing perioperative management and minimizing the risk of ischemic injury during carotid revascularization procedures. Preoperative assessment of autoregulatory function and collateral capacity may help identify high-risk individuals and guide tailored intervention strategies to mitigate these risks.

**Figure 1:**
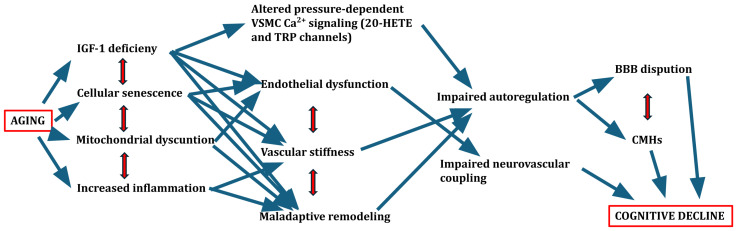
Schematic figure of age-related changes affecting the regulation of cerebral blood flow and possible consequences.

In addition to these vascular and surgical contexts, traumatic brain injury (TBI) represents another clinical scenario where impaired cerebral autoregulation has critical consequences [120], particularly in the aging brain [121]. Older adults are at increased risk of TBI due to higher rates of falls and greater cerebral vulnerability associated with vascular aging, brain atrophy, and comorbidities [122]. Following TBI, cerebral autoregulation is frequently disrupted, impairing the brain’s ability to maintain stable CBF in response to fluctuations in systemic blood pressure [123]. This loss of autoregulatory control exacerbates the risk of both secondary ischemic injury and hyperperfusion-related damage, contributing to poor neurological outcomes [124]. Importantly, age-related deterioration in baseline autoregulatory capacity further amplifies this vulnerability in older TBI patients [122]. Studies have shown that impaired autoregulation after TBI is associated with larger lesion volumes, greater BBB disruption, and an increased burden of cerebral microhemorrhages—pathological features that are already prevalent in the aging brain [121,125]. Moreover, the combination of TBI-induced and age-related cerebrovascular dysfunction may synergistically promote cognitive decline and neurodegeneration [126]. Given these observations, early assessment of autoregulatory function and careful management of blood pressure are essential components of TBI care, particularly in older adults, to mitigate secondary brain injury and improve clinical outcomes [127]. The mechanism described is summarized in Figure 1, which schematically illustrates the age-related changes affecting cerebral blood flow regulation and their potential consequences.

## 5. Methodological Considerations

Noninvasively assessing cerebral autoregulation in older adults presents methodological challenges [128,129,130,131,132,133]. Techniques such as transcranial Doppler (TCD) ultrasound, arterial spin labeling (ASL) MRI, and near-infrared spectroscopy (NIRS) provide non-invasive estimates of CBF and autoregulatory function but differ in applicability across populations. Selecting the optimal modality requires balancing temporal resolution, spatial coverage, invasiveness, logistical demands, and susceptibility to technical pitfalls. Accordingly, TCD excels at real-time assessment of cerebral haemodynamic responses, which makes it ideal to assess dynamic cerebral autoregulation in neurointensive units [133,134]. NIRS provides continuous, non-invasive and operator-independent measurements, being ideal to assess, for example, delayed ischemic changes by the bedside [135,136]. ASL offers quantitative, global perfusion maps, having the highest spatial resolution among the non-invasive methods [137,138]. Phase-contrast MRI quantitatively measures vessel-specific flow and pulsatility, enabling for regional assessment of autoregulation [139,140]. In conclusion, no single modality is universally superior, each reflects answers a different aspect of the autoregulation puzzle. Integrating their complementary strengths—rather than substituting one for another—yields the richest, most reliable haemodynamic assessment of the injured or at-risk brain. It has to be noted that cost-effectiveness should be considered when planning monitoring of autoregulatory function in older individuals. The below comparative table (Table 1) describes these aspects in detail.

Blood–brain barrier disruption and increased permeability are important consequences of autoregulatory impairment [141,142]. Different modalities provide data on distinct aspects of BBB function and molecular transport. Dynamic contrast-enhanced (DCE) MRI measures the rate of movement of contrast from plasma to extravascular extracellular space, its volume fraction and the rate of contrast reflux back to plasma, providing parallel, high-resolution data on perfusion and permeability [143]. ASL MRI techniques can be adjusted to assess perfusion and permeability alike, by using magnetically labelled water molecules as tracers and quantifying their exchange between compartments, obviating the need for intravenous contrast administration [144]. PET tracers may only cross into the parenchyma by specific transport mechanisms, capturing the integrity of the BBB at the molecular level [145]. These methodologies should be applied complementarily to unveil the pathobiology of permeability changes in detail.

The presence of common age-related comorbidities, including hypertension, diabetes, and atherosclerosis, further complicates the interpretation of autoregulation measurements, highlighting the need for careful study design and analysis. For example, chronic hypertension thickens arteriolar wall, shifting autoregulatory breakpoints [146,147]. Type-2 diabetes induces endothelial dysfunction and impairs nitric-oxide signaling, narrowing the plateau, and increasing inter-beat flow variability [148,149]. Accordingly, cerebral-autoregulation research therefore demands comorbidity-aware recruitment and analytic models that incorporate vascular phenotype as an interactive covariate.

Longitudinal, population-based studies tracking the decline of autoregulatory function with aging are scarce and contradictory [6,150,151,152,153]. For example, Weijs et al. showed that in a relatively small group of patients that a decade of aging did not lead to deterioration in CBF or autoregulation, while reductions in CBF and increases in cerebrovascular resistance were associated with early subjective cognitive decline [128]. Contrary, the MOBILIZE Boston Study showed that in 96 participants impaired autoregulation was obtained at baseline (indicated by higher transfer function gain), which was associated with greater decline in gait function during a 7 years follow up [153]. The longitudinal findings reported by Weijs et al. and the MOBILIZE Boston Study present seemingly conflicting conclusions regarding age-related changes in cerebral autoregulation and their clinical implications. Weijs et al. observed, in a relatively small cohort, that a decade of aging did not result in significant deterioration of cerebral blood flow or autoregulatory function. Instead, their data indicated that reductions in cerebral blood flow and increases in cerebrovascular resistance were more closely associated with early subjective cognitive decline. In contrast, the MOBILIZE Boston Study, encompassing a larger sample of 96 participants, documented impaired cerebral autoregulation at baseline, as evidenced by elevated transfer function gain, which correlated with greater decline in gait function over a seven-year follow-up period.

These divergent results may be reconciled by careful consideration of methodological and population differences between the two studies. First, the sample sizes differ markedly, with the MOBILIZE Boston Study having greater statistical power to detect subtle autoregulatory impairments and their functional consequences. Second, the study designs differ; Weijs et al. employed a longitudinal approach with a small sample concentrated on cerebrovascular parameters and cognitive outcomes, whereas MOBILIZE incorporated broader functional assessments including gait, suggesting differential sensitivity to cerebrovascular changes. Third, participant comorbidities vary; the MOBILIZE Boston cohort likely included greater heterogeneity in vascular risk factors and baseline clinical status, which may augment autoregulatory dysfunction and its downstream effects. Lastly, the metrics employed differ: Weijs et al. focused primarily on cerebral blood flow and cerebrovascular resistance measures, while the MOBILIZE Boston Study utilized transfer function analysis of cerebral autoregulation and linked these measurements to motor function decline.

Together, these distinctions underscore the complexity inherent in measuring cerebral autoregulatory function longitudinally, particularly in aging populations with varying baseline risks. They highlight the necessity for large-scale, multimodal, prospective studies incorporating comprehensive assessments of cerebrovascular physiology, comorbid burden, and clinically relevant functional outcomes to clarify the trajectory and clinical significance of age-related changes in cerebral autoregulation [128,153].

Although, cross-sectional studies reported increased arterial stiffness and reduced vasomotor activity associated with hypertension and atherosclerosis, study design limits the ability to distinguish age effects from disease-related changes [154]. These results highlight the need for further multimodal prospective research to assess age-related changes in autoregulation and its consequences on brain function. Especially, that animal models of cerebrovascular aging, while informative, often fail to fully recapitulate the complexity of human vascular aging, underscoring the translational gap in the field.

## 6. Potential Interventions and Future Directions

Lifestyle interventions that preserve vascular health, such as regular aerobic exercise and adherence to dietary patterns like the Mediterranean diet, hold promise for mitigating age-related cerebrovascular dysfunction. Evidence suggests that such strategies can improve endothelial function and promote cerebrovascular resilience [155]. The role of Mediterranean diet in preventing age-related alterations in the regulation of CBF should be tested in the future. It is increasingly recognized that sex-specific mechanisms play a critical role in vascular aging [156] and may influence both the trajectory of cerebrovascular decline and the response to preventive interventions. Differences in sex hormones [157,158], genetic factors, and vascular biology contribute to distinct patterns of endothelial dysfunction, vascular stiffness, and CBF regulation between men and women as they age [159]. These differences underscore the need for sex-specific considerations [160,161] when designing and evaluating lifestyle interventions aimed at preserving cerebrovascular health. Moreover, epidemiological studies aimed at understanding the determinants of cerebrovascular health across the lifespan [162] are essential for identifying modifiable risk factors and potential targets for intervention [163]. Such studies can provide critical insights into how lifestyle, metabolic, and environmental factors interact with biological aging processes to shape cerebrovascular outcomes. Future studies are warranted to test how complex lifestyle interventions impact autoregulation of CBF in older adults.

Pharmacological interventions targeting the molecular hallmarks of vascular aging represent an exciting, yet largely exploratory, avenue [164,165]. Various anti-aging treatment regimens [166], including antioxidants [167], mTOR inhibitors [168], and senolytics [158,169] that selectively eliminate senescent cells are under investigation for their potential to rejuvenate vascular function, including cerebral autoregulatory capacity.

Future research should prioritize interventional studies to determine whether improving vascular health translates into restored autoregulation and cognitive protection in older adults. Integration of vascular aging biomarkers into personalized risk stratification frameworks could further guide prevention and intervention strategies.

## 7. Limitations

There are several factors that may influence age-related alterations in cerebrovascular autoregulation, including sex differences, ethnic and racial disparities in cerebrovascular aging, sleep and sleep disorders, COVID-19 and its long-term vascular and endothelial sequelae, computational modeling of autoregulation, and medication effects (such as antihypertensives, statins, vasoactive drugs, and dementia subtypes). However, quantifying the impact of these factors is beyond the scope of the present manuscript but could provide valuable directions for future research.

The role of IGF-1 deficiency in pathophysiological changes in the regulation of cerebral blood flow has been suggested by animal studies. Although we recently demonstrated that IGF-1 deficiency is associated with impaired neurovascular coupling responses in older individuals, further clinical studies are needed to examine the role of deficient IGF-1 related signaling in age-related alterations of regulation of CBF.

## 8. Conclusions

Aging impairs cerebral autoregulation through a complex interplay of structural, functional, and molecular mechanisms. The resulting instability in CBF contributes to increased vulnerability to ischemic injury, cognitive decline, and poor outcomes following cerebrovascular insults. Preserving cerebral autoregulatory function is a critical but underappreciated target for promoting healthy brain aging. A concerted research effort is needed to elucidate the mechanistic underpinnings of autoregulatory decline and to develop targeted interventions capable of maintaining or restoring cerebrovascular resilience in older adults. Based on this, a clear and easy to follow clinical algorithm should be developed for risk stratification, methodological modality, interpretation and management.

## Figures and Tables

**Table 1:** Comparison of non-invasive methods used to assess different aspects of cerebral autoregulation.

Modality	Metric	Strengths	Limitations	Best Application
TCD	cerebral blood flow velocity (cm/s) in major intracranial arteries and derived autoregulatory indices	bedside applicability high temporal resolution	poor spatial resolution interobserver variability requires bone window	continuous bedside monitoring of dynamic autoregulation in an ICU setting
NIRS	TOI (tissue oxygenation index) TOx (moving correlation coefficient of TOI and MAP, with positive values indicating impaired, and negative values reflecting maintained autoregulation)	bedside applicability high temporal resolution real-time regional autoregulatory information	limited penetration depth mainly cortical measurements	continuous, bedside autoregulatory monitoring with excellent temporal and acceptable spatial resolution
ASL	regional perfusion (mL/100g/min)	non-contrast perfusion maps high spatial resolution	poor temporal resolution, not applicable for dCA sensitive to motion artifacts	monitoring of functional hyperemia (neurovascular coupling)
PC-MRI	vessel-specific flow (mL/min) and pulsatility quantification	quantitative blood flow measurement in specific major arteries	limited temporal resolution, not applicable for dCA susceptible to motion artifacts	vessel-specific metrics of flow and pulsatility

TCD—transcranial Doppler, NIRS—near-infrared spectroscopy, ASL—arterial spin labeling, PC-MRI—phase-contrast MRI, dCA—dynamic cerebral autoregulation.

## Data Availability

Data sharing not applicable.
